# Hospital and Institutional News

**Published:** 1914-03-14

**Authors:** 


### HOSPITAL AND INSTITUTIONAL NEWS.

### "THE HOSPITAL'S" SPECIAL NUMBER ON RADIUM.

Next week's issue of The Hospital will comprise a Special Number, which will be devoted to the subject of radium, its cost, therapeutic value, quality, and storage, which are now causing so many heart-searchings in the institutional world. An immense amount of expert information has been collected, and The Hospital's Special Number next week will form a unique monograph, embodying the latest practical knowledge obtainable on the four aspects mentioned above. While institutions of every class are bidding against each other, and the mayors of several centres of population are considering the propriety of inaugurating a special Radium Fund, while the medical officers of Poor-Law infirmaries are pressing on Boards of Guardians the need for adding this substance to their equipment, the moment is ripe for presenting both the commercial and therapeutic facts which govern radium at the present time. We believe in this manner that The Hospital will be rendering a much-needed service to the institutional world. The Special Radium Number will be issued, as usual, at the price of One Penny.

### RADIUM FOR THE WITHINGTON WORKHOUSE HOSPITAL.

The question of the supply of radium at Poor-Law infirmaries has been under consideration by various Boards of Guardians. In London, at St. George's-in-the-East Infirmary, a small quantity has been available for some time past, owing to the kindness of a friend of the medical superintendent; and the Wandsworth Guardians passed a resolution in October of last year that the Local Government Board should be asked to authorise the establishment of a department under the control of the Metropolitan Asylums Board for the supply of radium to the various infirmaries in the Metropolitan area. This resolution was sent to all the London Boards of Guardians, and met with a certain amount of support, but so far nothing further has been heard of it. The South Manchester Guardians have recently discussed the matter, and have decided, on the advice of Mr. Wrigley, the visiting surgeon to the Withington Workhouse Hospital, to ask the Local Government Board for sanction to borrow money to obtain a supply of radium for the hospital. Mr. Wrigley reported that there are at present twenty-two cases of inoperable cancer in the hospital, some of which were suitable for radium treatment, and that during 1913 123 cases of malignant disease had been admitted, the great majority of them inoperable and some of them of a character likely to benefit from the use of radium. The action of the Local Government Board will be awaited with interest, and if the South Manchester Board are successful in their application, other Boards will probably follow their example.

### A LEGACY FOR THE ASYLUMS BOARD.

We drew attention on February 15, 1913, to the curious fact that, under the will of Mrs. E. R. Johnson, three sums amounting to £7,000 were bequeathed to three fever hospitals under the control of the Metropolitan Asylums Board. The proposals to use the money in relief of rates, and to apply it to the voluntary hospitals, since it is assumed that the testator imagined that the Board's hospitals were charitable institutions, were defeated, and an amusing correspondence followed in The Hospital, in the course of which various suggestions for the employment of the legacy were made. Since then, on the suggestion of the Local Government Board, the Charity Commissioners have been consulted, and last Saturday the Finance Committee of the Board submitted a formal application to the Commissioners which consisted of three proposals. First, that the managers should be appointed trustees of the Johnson legacies; second, that the principal sums should be invested in trust securities; and, lastly, which is the main point of interest, that the income be applied to provide "means of recreation" for all whose illness or employment lead them to be occupants of the North-Eastern Hospital, the Northern Hospital, and the Southern Hospital, the three beneficiaries which received £1,000, £2,000, and £4,000 respectively. This seems a satisfactory decision, but everything depends on how it is carried out. Means of recreation may mean anything from a billiard-room to a tennis court; and the question is complicated by the fact that the staff and the patients are both to enjoy the added facilities. We invite suggestions from the staffs and patients of the institutions referred to, which would at least provide an amusing correspondence, and would probably, if sufficiently unanimous, lead to the adoption of the proposals.

### THE ST. ALBANS AND MID-HERTS HOSPITAL.

At the annual meeting of this hospital recently held, Mr. C. T. Part, the chairman, mentioned our criticisms which pointed out the imperative necessity for improving the kitchen and larder arrangements, and also certain sanitary arrangements. He does not appear, however, to have read the report on this institution which was published in The Hospital of February 21, page 559, for Mr. Part contented himself with saying they had tried in all sorts of ways to improve the defects in the buildings referred to without being able to arrive at any satisfactory solution. May we assure Mr. Part that all that is required are the services of a competent hospital architect who should be able, as we satisfied ourselves when we inspected this hospital, readily to produce a plan providing for the necessary reconstruction which would materially improve the appearance, and the working and hygienic conditions of the hospital as it exists to-day. We commend this matter to the attention of the *Herts Advertiser*, the editor of which in August 1913 declared that, whatever might be the standard of administration of voluntary hospitals in other cathedral cities, the *Advertiser* preferred to regard the St. Albans and Mid-Herts Hospital as exceptionally efficient and certainly not out of date.

### DOMESTIC SERVANTS AS IN-PATIENTS.

A long and interesting discussion took place at this meeting in regard to the use of the St. Albans and Mid-Herts Hospital by domestic servants. The existing rules provided that a charge of 12s. 6d. per week should be made in respect to such persons admitted as in-patients. The committee favoured the abolition of this rule, but the governors, by a small majority, upheld the old rule on the ground that the hospital is intended for poor people, and that domestic servants, being as a rule very well paid, can properly contribute towards the cost of their treatment as in-patients. It was a great convenience to employers to use the hospital for their servants, and they ought to contribute towards the extra 2s. 6d. or 5s. a week due to the hospital in addition to the sickness benefit received under the Insurance Act. The report, the attendance at the annual meeting, on which we congratulate the committee, and the animated discussions prove the warm interest taken in the St. Albans and Mid-Herts Hospital by all sections of the governors.

### SMALLER HOSPITALS AND THE MODERN STANDARD.

The proceedings at the annual meeting of the West Norfolk and Lynn Hospital reveal the reason why some of the smaller and older voluntary hospitals are not up to the modern standard. Mr. G. E. Rose made the practical proposal that a Finance Committee be elected each year of independent governors, with the object of attending strictly to the business side of and actively working to secure an adequate income for the institution. It was urged that such an independent committee might come into conflict with the Monthly Board, a statement which reveals to those accustomed to the government of an actively administered voluntary hospital the need there is for development and knowledge amongst the governors. A Finance Committee of five members, being all of them good men of business, would strengthen the Monthly Board, of which they would necessarily be *ex-officio* members at any rate. Such a committee would report regularly to the Monthly Board, and act in co-operation with it. This essential improvement in the existing system of management was, however, negatived in favour of Sir Somerville Gurney's suggestion that the old Finance Committee, consisting of the whole of the Weekly Board, should be reappointed. Of this body the Rev. E. W. Bremner, an old member, declared it was not in the true sense a Finance Committee at all. If it were a real Finance Committee, men might be willing to serve upon it. We fear, therefore, there is no hope that reform and progress will make headway at this institution under present circumstances. Yet the *Eastern Daily Press*, which we hoped would make an independent stand on behalf of the hospitals of Norfolk being brought up to the standard of up-to-date, modern institutions, declares in a leading article in its issue of February 27 that on the whole the authorities of this hospital have made good their reply to the criticisms on the institution as it exists to-day. Yet there is no more urgent and important matter to the inhabitants of the district throughout which the *Eastern Daily Press* circulates than the up-to-date efficiency of every hospital situated within its area.

### A HOSPITAL OFFICER'S JUBILEE.

Sir George Hare Philipson, who is celebrating his jubilee as a medical practitioner, has devoted the greater part of his time to the Royal Victoria Infirmary, Newcastle. It is, in fact, fifty years since he became connected with the old institution out of which the present hospital has grown. So long ago as 1864 he became associated with the pathological department, and four years later he became a member of the honorary medical staff. Perhaps the most notable feature of this period was the fund raised eighteen years ago by Sir Riley Lord to build the existing institution, and the passage of time has brought about the need for raising a similar sum in order to extend it. It is a wonderful development to have lived through and to look back upon, and we hope that Sir George may see also the much needed extension of the infirmary, the reduction of its waiting list, the removal of the expediency of additional beds, and the further £10,000 a year which will be needed for its maintenance.

### A NEW FACTOR IN HOSPITAL DESIGNS.

It is pleasant to hear from Sir G. Wyatt Truscott, Bart., treasurer, City of London Hospital for Diseases of the Chest, that the feature of the festival in December was the great support given to the hospital by the working-men's societies of the East End of London. The total of such subscriptions well exceeded £1,000, an amount which comes from the class which benefits most from the institution. During the past five or six years the hospital has been renovated throughout, and the old deal boards of the wards have been replaced with polished teak. In addition to internal improvements, a sanatorium, in connection with the hospital, is being provided at Saunderton, in Buckinghamshire, under the care of the Ladies' Committee, who have purchased a site of some fifty acres. The plans of the building have been supervised by Dr. Glover Lyon from the medical standpoint, and within the year it is hoped to have the sanatorium completed. It is proposed to have two blocks—one for men, the other for women. At last week's annual meeting an interesting sidelight on current affairs was revealed by Mr. Herbert Robertson, who said that a number of alterations had been found necessary to the existing hospital, including further baths and a boilerhouse, whereby space would also be found for increased coal storage, especially so as to be able to tide over labour troubles. It is curious to note how hospital construction may be modified by events apparently so remote from its design as industrial disputes.

### NEWCASTLE WORKMEN AND THE TRUCK BILL.

IN view of the immense support given by working-men to the Royal Victoria Infirmary, Newcastle-upon-Tyne, the resolution lately passed unanimously by the governors disapproving of the proposal to amend the Truck Acts so as to prevent deductions from wages in the form of subscriptions to hospitals is highly significant. The working-men, we learn on the highest authority, are not only willing that their contributions should be deducted at the pay offices, but actually ask the employers to do it as a favour. The local belief is that " such a gratuitous piece of interference as the introduction of the word ' hospital ' in this Bill " is not due to an oversight, but that there is some truth in the argument that the movers of the Bill desire to cripple the voluntary hospitals so as to drive them into the hands of the State. This result certainly would not be achieved, and we gather that there is little chance of this Bill, as it stands, making much further progress.

### MIGRATION OF THE WEST END HOSPITAL.

THE latest hospital in London to move, not however from the centre to the periphery of the Metropolis, is the West End Hospital for Nervous Diseases, which, Mr. John Brassey announces, has decided to leave its present quarters in Welbeck Street and to build a new institution either on a site in Northumberland Street, Marylebone, or in Vincent Square. On general principles we favour the latter, and, should it be decided on, Vincent Square will have two hospitals at this address practically built within twelve months. The proposed institution is expected to cost 40,000*l.*, and it is interesting to recall that the present hospital, which was established in 1878, was rebuilt in 1891, and at the present moment possesses forty-two beds.

### A JOINT SANATORIUM FOR BERKS AND BUCKS.

THE County Council of Berkshire has lately contemplated the possibility of using the existing sanatorium accommodation at Peppard known as the Maitland Sanatorium, with the adjoining property, Kingswood. The number of adult beds available being in excess of that required by Berkshire, negotiations were opened with Buckinghamshire, and a conference has been held with representatives of their Public Health Committee, who unanimously decided to recommend the desirability of becoming partners with Berkshire in a scheme for utilising these institutions. Options to purchase during the three months ending March 31, 1914, have been obtained in respect of both the Maitland and the Kingswood properties, and in the event of a combined scheme being approved, a joint committee on which both County Councils will be represented will manage the institutions. The Maitland Sanatorium has been managed by Mr. H. F. Carling, as superintendent, and his wife, Dr. Esther Carling, as resident medical officer. The adjoining property, Kingswood, is carried on at a personal profit by Dr. Esther Carling. The trustees have offered to sell outright the Maitland site and buildings for £8,500, and Dr. Carling has offered to sell the Kingswood site for £5,000 provided the services of herself and Mr. Carling are retained for five years. The Charity Commissioners are prepared to approve a sale of the trust, and the Board of Education will probably approve of the site as one suitable for the treatment of tuberculous children. This latter provision will be only temporary, for it is almost certain that in the future the accommodation for sanatorium treatment for children will require considerable extension, and necessitate the erection of a residential school, in which the children will receive special instruction in manual and outdoor work whilst undergoing treatment.

### ILLNESS OF THE MEDICAL SUPERINTENDENT OF CROYDON INFIRMARY.

WE regret to hear that Dr. R. W. Wilson, the medical superintendent of the Croydon Poor-Law Infirmary, is suffering from septicæmia, contracted from a patient upon whom he operated last week. Symptoms set in very shortly after operation, and became so serious that Dr. Wilson was sent to St. Mary's Hospital, Paddington. We are glad to hear that he is improving, and that at the time of writing his condition is satisfactory.

### THE RECONSTRUCTION OF THE BROWNLOW HILL WORKHOUSE.

IN THE HOSPITAL of January 1, 1910, Sir Henry Burdett drew attention to the scandalous condition of the sick wards of the Brownlow Hill Workhouse. Since then various improvements have been introduced, but it has been patent to all acquainted with the facts that nothing short of a drastic reconstruction of the building could meet the position. This has now been brought a step nearer realisation by a Memorandum of the Local Government Board upon the condition of the building and the changes required to make it satisfactory. The Memorandum fully bears out Sir Henry Burdett's criticism of four years ago. It states that: " The workhouse and infirmary buildings at Brownlow Hill combine together to form a mixed workhouse of an obsolete type, in which adequate classification is impossible, and of which the main imperfections may be summarised as arising out of (*a*) an insufficient site; (*b*) a congested and haphazard arrangement of buildings; (*c*) buildings themselves of an antiquated type; (*d*) lack of open ground; (*e*) danger of fire; (*f*) defects of heating, drainage, etc. We suggest that the number of persons accommodated upon the workhouse site at present is much in excess of what is reasonable or hygienic." The Memorandum proceeds to point out that the density of the population upon the workhouse site is 279 persons per acre when the accommodation is fully taken up, and that in times of stress this density reaches still higher figures, as compared with a density of population of 45 persons per acre for the whole of the city and of 112 persons per acre for the centre of the city!

### A BLOT ON POOR-LAW ADMINISTRATION.

The report contains a well earned encomium of the medical officers of the institution for "the patient and resourceful manner in which they have contended with the administrative and other difficulties arising out of the defects and shortcomings of the buildings." The Local Government Board suggests that a miniature town-planning scheme for the area should be prepared, under which the whole site should be gradually developed as an infirmary for acute cases with about 1,200 beds; that is, for rather less than half of the population at present accommodated. If this scheme is adopted, the Highfield Infirmary might be used for chronic and convalescent cases and for sick children, and the Kirkdale institution as a workhouse for healthy inmates. Reform has been a long time coming, but if the Liverpool Select Vestry adopt the recommendations of the Local Government Board and energetically push forward their realisation one of the great blots on Poor-Law administration will soon be a thing of the past.

### THE NEW MIDDLESBROUGH SANATORIUM.

The provision for the treatment of tuberculosis which has been made by the Middlesbrough Corporation includes not only a dispensary where out-patients are treated, the sanatorium at Hemlington, but also an institution in West Lane for advanced cases. Known as the West Lane Observation Hospital, and opened by the Mayor in the presence of Alderman J. McLauchlan, chairman of the sanatorium committee, the new institution contains accommodation for twenty-eight patients, divided equally between the sexes, and the Insurance Committee is sending six of their patients at a weekly cost of twenty-five shillings. The new institution, however, is chiefly interesting as marking the completion of one of the most comprehensive schemes which a municipality has yet carried out for dealing with tuberculosis. At the two institutions sixty in-patients are provided for, and the cost of the three has been £4,744, for which the Government grant has been £2,184 in aggregate, or nearly half the expenditure. About two-thirds of the maintenance expenses will be provided for by the Exchequer's and Insurance Committee's contributions, leaving, according to present estimates, just under a penny in the pound to be provided by the rates. The above is noteworthy as showing what can be undertaken in this direction by a municipality which really sets to work on this question, and three institutions for £5,000, if they are well constructed, is not an extravagant sum to pay for comprehensiveness and classification.

### THE NIGHT STAFF AT POOR-LAW INFIRMARIES.

An inquest was held last week into the death of an inmate of Hackney Infirmary, whose death was accelerated by a fall out of bed. The fall occurred during the night, when the ward, which contained twenty-one patients, was in charge of a probationer who had had only three months' nursing experience. Despite the evidence given that the ward was visited frequently by the night superintendent—a fully trained and experienced nurse—and that she and the medical staff were within easy call, the jury added a rider to their verdict that a ward of this size ought to be in charge of a fully trained nurse both by day and night. The verdict illustrates how the standard by which Poor-Law infirmaries are judged by the public is increasing. There is probably no Poor-Law infirmary in England where the wards are in charge of fully trained nurses at night, and, speaking generally, there is no reason why there should be. In most infirmaries the average number of beds in a ward is about thirty, and in most of them one probationer is in charge of the ward during the night, except in the case of the surgical or children's wards, when the night staff often consists of two probationers. In the case under review, therefore, the number of patients under the charge of the probationer compares favourably with the conditions at other infirmaries. It must be pointed out, however, that it is unusual to place a probationer with only three months' experience in a position of this responsibility. It is more usual not to place a probationer upon night duty until she has had at least six months' experience.

### PUBLIC OPINION AND POOR-LAW STANDARDS.

The Hackney Infirmary has about 760 beds and only one night superintendent. This is certainly inadequate. In an infirmary of this size there should be at least three night superintendents. It is interesting to note the effect public opinion has had upon the increase of the nursing staff under the Poor Law during the last thirty years. In 1883 the number of nurses in Poor-Law institutions was 2,385, in 1913 it was 7,652. That the staff, however, is still far from sufficient is shown by the fact that the number of patients to each nurse in the London infirmaries varies between six to twelve patients per nurse. Comparing this with the average of one nurse to 2.33 patients obtaining in the large London hospitals with medical schools, and, making all allowance for the differences in the type of patients admitted to the two classes of institutions, one sees that much still requires to be done before the Poor-Law nursing staff can be considered to meet the requirements of to-day. To obtain efficient nursing in an average Poor-Law infirmary a staff of at least one nurse to five occupied beds is required, and of this staff not more than 30 per cent. should be probationers of less than one year's experience.

### A COTTAGE HOSPITAL AND PUBLIC AUTHORITIES.

The Passmore Edwards Cottage Hospital at Acton complains in its latest report of unfavourable treatment by the National Insurance Commissioners and by the Urban District Council. The report, to which Mr. E. F. Hunt, honorary secretary to the institution, is one of the signatories, states that as "the hospital gives first aid to insured persons, and assists and relieves them, it is entitled to some payment for this work." There is no doubt that but for the institutional treatment provided by the voluntary hospitals the Insurance Act would have broken down completely, but we should be glad to know what door Mr. Hunt thinks was left open in the Act itself by which the Insurance Commissioners could be approached officially by hospital managers. In the case of the Urban District Council it should be easier to press the hospital's claims, for the report states that they have terminated a hitherto existing agreement under which the hospital was paid £50 per annum in return for accommodation reserved for the Council's workmen. As no reason is given why the Council have cancelled this arrangement it looks as if they were merely seeking an excuse to withdraw their support, irrespective of the valuable public health work which every efficient hospital performs for its neighbourhood.

### FOR HOSPITAL WORKERS.

It is our purpose to make each number of The Hospital of practical use and interest to hospital workers in every department of institutional service. We could wish that the secretaries and matrons would direct the attention of their staff in the various departments to the Institutional Worker section of The Hospital. In that section our aim is to exchange information with all institutional workers. A reference to its pages will show that during February it has contained practical information on contracts for supplies; ward furniture, with some useful illustrations of new types of equipment; original designs for coal bunkers, with full descriptions and illustrations of more than one kind; the comparative values of meat, with full details, and a comparison of the relative nutritive qualities of foreign and English meat; and a portrait of a hospital worthy, for thirty-seven years in responsible charge of the commissariat of the same hospital. Every post brings us a number of inquiries on practical points, showing the need and value of the Institutional Worker section. We want to enlarge it and make it useful to each worker, in whatever department of a hospital or institution he or she may be employed. The co-operation of all such workers and of the responsible officers of the institutions generally is therefore invited to enable this department of the paper to attain its maximum usefulness. Will the more practical of our readers keep an eye on this section and help us with a little personal service by making it known to their staff? The columns for questions invited and questions to be answered present an opportunity for co-operation which, if availed of, may result in many advantages to the individual.

### THE RIVER AMBULANCE SERVICE.

An innovation which has been carried out quietly by the Metropolitan Asylums Board has proved so far a success. The Board has instituted a fleet of six steamers to be used for the carrying of patients to their isolation hospital at Deptford. Each steamer is fully equipped to deal with the immediate needs of the patients, who number from twelve to fourteen, and is in charge of a fully-qualified nurse. Since October over 5,000 patients have been conveyed from Long Reach to Dartford by the steamers. The journey by river is accomplished in an hour and a quarter, whereas by road it takes over four hours, which alone is an important factor in favour of the new service. The introduction of the "steamer ambulances" are due to Captain Thimm, R.N., a member of the Metropolitan Asylums Board, who for some years was in charge of the river ambulance service at Rangoon during the plague in India. He is chairman of the sub-committee controlling the river ambulances, and his experiences in India have been of considerable assistance in helping to make the new service a success. It appealed strongly to the Board when it was first suggested, inasmuch as it was held that it would be a great saving, an anticipation which so far has been justified by the result.

### IRELAND AND VENEREAL DISEASE.

The appointment of the Royal Commission on Venereal Diseases was symptomatic of a change in public opinion on this question, on which a sidelight is thrown in the evidence of Mr. Ballance, chief surgeon of the Metropolitan Police, at the twentieth meeting. In the course of his evidence he stated that his experience at St. Thomas's Hospital, to which he is surgeon, indicated that there were fewer serious cases than formerly, a fact probably accounted for by earlier treatment, which was replacing the neglect of bygone days. That this change of view can hardly be said to exist in Ireland is not surprising. Dr. Brian O'Brien, medical inspector of the Local Government Board, who gave evidence at the twenty-first meeting, declared that the treatment of venereal disease in Ireland was most inadequate. It was specially prevalent in Dublin, which he described as a focus of undesirables, and poverty and bad housing had a large responsibility for this. We thus see, in this parallel evidence from the two countries, the relative value of attention and neglect, and as attention in England has led to the formation of the Commission we can only hope that its proposals will be made to apply to Ireland, whereby its boasted "purity" may have the additional safeguards which public attention to the risk of venereal infection can provide.

### FINANCE AND DEVELOPMENTS AT BRIGHTON.

The retirement of Mr. C. R. Scrase-Dickins from the chairmanship of the Royal Sussex County Hospital has practically coincided with the need for further development, for which a sum of £15,000 is required to centralise the heating and hot-water services and to extend the nurses' home, which, thanks to the loan of a flat, has remained the same size as it was when the beds were 140. They are 201 at the present day. It is seven years since Mr. Scrase-Dickins was appointed chairman, and his successor in that office, Mr. Ball Dodson, notes the curious fact, which is surely worth an explanation, that the total expenditure last year was almost precisely the same as in 1906, when the daily average number of occupied beds was only 162; it is now 191. It is not enough to congratulate the governors on the greater benefit apparently thus derived from the same expenditure; the new economies or the old extravagances should be pointed out. An explanation of this sort, which must be perfectly understood by the Secretary, Mr. R. B. Jay, is likely to be sought by intelligent testators who, now that there is a Royal West Sussex as well as a Royal Sussex County Hospital, are likely to make careful inquiries owing to the possibility of a confusion of names.

### THE SALARY OF INFIRMARY SUPERINTENDENTS.

Dr. H. W. Bruce, medical superintendent of the Southwark Infirmary, has been rewarded for his services by a modest increase in his salary of £50 a year, bringing it up to £500. He was appointed to the position in 1905, being formerly assistant medical officer, and his labours were so well recognised by the Southwark Board of Guardians that the increase was granted him without discussion. The infirmary committee reported that he had "invariably carried out his important duties most satisfactorily." Last summer, as our readers will remember, Dr. Bruce had to deal with a serious outbreak of enteritis amongst the children in the infirmary, due, in his opinion, to the close proximity of the refuse siding belonging to the Camberwell Borough Council. The Local Government Board took the matter in hand, and decided to call upon the Council to close the siding. The action taken by Dr. Bruce has earned for him the gratitude of both the infirmary and the many residents living in the thickly populated area in close proximity to the siding, and their only desire is that his efforts to remove a public nuisance will be met with success.

### GUARDIANS AND MEDICAL CERTIFICATES.

The attempt made by various Boards of Guardians to prevent their medical officers from giving medical certificates under the Insurance Act to the sick under their charge has received another blow as the result of a question asked by Mr. Locker-Lampson in the House of Commons. The Marylebone Board of Guardians resolved that medical certificates should not be given to the insured inmates of the infirmary unless the consent of the relief committee had previously been obtained. In one case which was referred to the relief committee under this resolution a certificate was refused, and the patient, in consequence, was unable to obtain from his approved society the benefit to which he was entitled. Mr. Locker-Lampson asked the President of the Local Government Board whether any pressure could be brought to bear upon the approved society or the infirmary officials so that the patient in question might obtain the sick pay due to him. Mr. Herbert Samuel, in his reply, stated that it was the duty of the medical officer of the infirmary under the Local Government Board's regulations to give a certificate to the person on whom he is attending of the sickness of such person or the cause of his attendance upon him, and that he was calling the attention of the Guardians to this requirement.

### EFFECT OF MR. SAMUEL'S PRONOUNCEMENT.

The result has been that the Marylebone Guardians have withdrawn their resolution. Mr. Samuel's authoritative pronouncement is an opportune one, and no doubt other Boards of Guardians, who have passed similar resolutions, will now adopt the same course. That both Boards of Guardians and medical officers have a grievance in the matter—the former owing to the difficulty they may experience in individual cases in obtaining any contribution towards their maintenance from insured persons, and the latter from the large number of certificates they may be required to give for Insurance purposes—is undoubted. Nevertheless, such grievances cannot justify the illegal withholding of certificates and some other remedy must be found.

### THIS WEEK'S DRUG MARKET.

Business has not been particularly active during the week, but there is nothing depressing in the tone of the market. Reports from the Norwegian fishing-grounds show further improvements in the results, and it is by no means improbable that at the end of the season the yield of oil will be larger than last season's, in fact, unless bad weather interferes with the fishing, this will almost undoubtedly be the case; prices for cod-liver oil are again lower, but prospective buyers are still disposed to delay their purchases in the expectation that the lowest figures have not yet been reached. The value of opium is well maintained, and the same can be said of morphine. Sulphonal is dearer. Strychnine is unchanged at the reduced price. Atropine has a dearer tendency. Essence of lemon is nominally unchanged in price, but the tendency seems to be in a downward direction. The values of tartaric acid and citric acid are well maintained. At the recent public auction of drugs in Mincing Lane the demand was rather better than of late, but there were few noteworthy changes in value. Asafetida was in plentiful supply, and a fair quantity sold at rates which showed little change. Coca-leaves were somewhat lower in price. East Indian ipecacuanha realised higher prices. Sarsaparilla was rather dearer. The value of honey was barely maintained. Jamaica wax was dearer.

### TO OUR READERS.

Contributions are specially invited from any of our readers to these columns. They should deal with topical subjects and news. They must be authenticated for the information of the Editor only. The minimum payment if published is 5s. There is no hard-and-fast rule as to space, but notes of about twenty lines in length are preferred.

